# Epistaxis management on COVID‐19–positive patients: Our early case experience and treatment

**DOI:** 10.1002/ccr3.3137

**Published:** 2020-07-21

**Authors:** Samba Siva R. Bathula, Tyler Patrick, Luxman Srikantha

**Affiliations:** ^1^ Department of Otolaryngology ‐ Head and Neck Surgery Detroit Medical Center Detroit MI USA

**Keywords:** COVID‐19, epistaxis, Merocel, surgicel

## Abstract

Epistaxis management on COVID‐19 patients is concerning for otolaryngologists due to the highly virulence and increased concentration within respiratory droplets and nasal secretions. Authors suggest initial management with oxymetazoline nasal drops and local pressure before considering nasal packing with absorbable material to prevent COVID‐19 transmission to surrounding healthcare workers.

## Graphical abstract

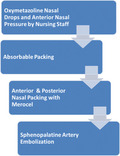

## 1 INTRODUCTION

Epistaxis is a common presenting symptom in the hospital setting and presents as a common consultation for otolaryngologists. It is known to be more severe in the adult population, especially those taking anticoagulant and antiplatelet medications.^1^ Unfortunately, with the exception of Pradaxa (dabigatran), there are no antidotes to stop epistaxis caused by newer anticoagulants such as Xarelto (rivaroxaban), Eliquis (apixaban), and antiplatelet medications such as aspirin, Brilinta (ticagrelor), or Plavix (clopidogrel bisulfate).^2^ Therefore, nasal packing with either nonabsorbable or absorbable material is a known effective treatment for epistaxis in the setting of anticoagulant usage.

The first novel coronavirus (also known as COVID‐19 or SARS‐CoV‐2) infection was identified in December 2019 in Wuhan, China, and spread throughout the world within a span of 2 months. The World Health Organization (WHO) on 11 March 2020 declared the COVID‐19 outbreak a global pandemic.^3^ COVID‐19 is highly contagious, and at this time, there is no effective treatment or vaccine available. Currently, recommendations to decrease transmission from Center for Disease Control and Prevention include social distancing, hand washing, and the use of PPE such as face masks and protective face shields.^4^

The COVID‐19 disease process includes multifocal pneumonia leading to respiratory failure; however, there are also new theories of microthrombi causing end‐organ damage.^5^ In our institution, this has led to more patients being placed on systemic anticoagulation, which has also led to an increase in the incidence of epistaxis.

Higher mortality has been reported in COVID‐19 patients with preexisting heart disease.^6^ This subset of patients often will subsequently be treated with the abovementioned anticoagulation and antiplatelet therapy. Additionally, patients often require supplemental oxygen via nasal cannula, which dries the nasal passages, making them prone for bleeding. Otolaryngologists are then called upon to manage acute epistaxis on COVID‐19 anticoagulated patients in the critical care and noncritical care settings.

The primary objective of this paper was to provide an algorithmic approach to epistaxis amid the COVID‐19 pandemic. The management in each case is described in detail along with a comprehensive discussion about absorbable and nonabsorbable nasal packing.

## 2 MATERIALS AND METHODS

This investigation is based on the retrospective chart reviews of four COVID‐19 PCR‐positive patients. All were treated in the hospital, either in an ICU or a medical floor, with prolonged epistaxis, and who were receiving active anticoagulant and/or antiplatelet medications. Specific focus during analysis of each patient's chart was placed on (a) clinical presenting symptoms, (b) results of the physical examination guiding all treatments rendered, and (c) final outcomes. All four patients were treated by otolaryngology junior and senior residents, under the supervision of an attending physician with proper protective equipment (PPE) including—gown, head cap, N‐95 mask, face shield, and gloves at the Detroit Medical Center. Bleeding was resolved within 48 hours of observation after nasal packing. The outcome was based on anterior and postnasal bleeding and determined by the attending physician.

## 3 RESULTS

Charts of four patients, who met the previously defined inclusion criteria, were comprehensively analyzed by the team of otolaryngology residents and the primary author who served as the attending physician (Samba Siva R Bathula, SSB). Electronic medical charts were also reviewed to assemble the data described below for each case.

### 3.1 Case 1(SSB)

A 58‐year‐old COVID‐19 PCR‐positive female patient who was intubated for respiratory failure and was admitted to the ICU. The patient had been hypertensive and was on aspirin 81 mg daily as well as enoxaparin 100 mg subcutaneous injections daily for deep vein thrombosis (DVT) prophylaxis. No coagulation studies (PT—prothrombin time, aPTT—activated partial thromboplastin time, INR—international normalized ratio) were noted in the chart on the day of bleeding but were normal 3 days before the bleeding. She began experiencing bleeding from the right nasal cavity after nasogastric tube (NGT) insertion for several hours. The primary ICU team had not tried any further interventions to mitigate epistaxis; ENT was consulted for further management. On physical examination, blood clots were noted in the bilateral nares as well as the oral cavity. The patient was neurologically unresponsive due to sedation for ventilation tolerance. Bilateral nares were packed with absorbable hemostatic packing (Surgicel® Fibrillar; Ethicon) in the posterior and anterior nasal cavities, and no further bleeding was noted after 48 hours.

### 3.2 Case 2(SSB)

A 58‐year‐old female patient, found to be COVID‐19 PCR‐positive, was intubated for respiratory failure in ICU and was on aspirin 81 mg daily and enoxaparin 100 mg subcutaneous daily for DVT prophylaxis. Slightly elevated PT (11.8) and INR (1.15) with normal aPTT were noted. Persistent slow nasal bleeding was noted by primary team following placement of a left‐sided NGT. Otolaryngologists placed bilateral Surgicel® Fibrillar packing to each nare. The left side was packed around the existing NG tube. The following morning, there was resolution of epistaxis. At this time, per nursing request, the NGT was removed and replaced with an orogastric tube. In this process, the left‐sided packing was removed to reveal scant nasal mucosal bleeding. The left nare was repacked with Surgicel® Fibrillar with resolution of bleeding. No further bleeding was noted after 48 hours.

### 3.3 Case 3(SSB)

A 55‐year‐old female COVID‐19 PCR‐positive patient who presented in an altered mental state was found to have an acute cerebrovascular accident as well as acute ST‐elevation myocardial infarction due to COVID‐19 coagulopathy. Home medication of aspirin 81 mg for a history of DVT was continued, and patient was placed a continuous heparin 25 000u IV + D5W 250cc drip. Elevated PT (12.9), INR (1.26), and aPTT (63.6) were noted. NGT was accidentally pulled by the patient, which caused nasal hemorrhage. ENT was called for continuous nasal bleeding of which was not controlled by oxymetazoline nasal drops and nasal pressure performed by nurse at bedside. Anticoagulation de‐escalation was recommended and heparin drip was discontinued. The entire left nasal cavity was packed with resorbable Surgicel® Fibrillar along with Surgicel® Original (Ethicon) and Surgifoam® (Ethicon). Patient remained stable, and bleeding resolved subsequently after packing. No further bleeding was noted even after 48 hours.

### 3.4 Case 4(SSB)

An 80‐year‐old COVID‐19–positive female patient was experiencing bilateral intermittent epistaxis. The patient was on Enoxaparin (Lovenox) 100 mg subcutaneous injections daily for DVT prophylaxis. Slightly elevated PT (11.9) and INR (1.16) with normal aPTT were noted. Verbal instructions were given to the treating nurse to provide oxymetazoline and anterior pressure, and epistaxis resolved. No further bleeding was noted even after 48 hours.

## 4 DISCUSSION

COVID‐19 is highly contagious and transmits from person‐to‐person through respiratory droplets, saliva, and blood. Otolaryngologists are particularly at risk of transmission because of the continual close proximity to secretions of the nose and throat. Standard personal protective equipment (N95 mask, surgical cap, face shield, surgical gown, and gloves) is therefore essential to examine COVID‐19–positive patients.

Epistaxis is one of the most common consults often managed by otolaryngologists. Epistaxis is divided into two subtypes: anterior and posterior. Anterior epistaxis most commonly originates from Kiesselbach's plexus and is often managed with several different methods including anterior nasal pressure, application of oxymetazoline nasal drops, anterior nasal septal cautery with either silver nitrate or electrocautery, and anterior nasal packing with either absorbable or nonabsorbable materials. Posterior epistaxis is typically managed with posterior nasal packing. If posterior packing does not resolve the bleeding episode, either ligation or embolization of the sphenopalatine artery can be considered.^7^, ^8^

Merocel® is a nonabsorbable nasal tampon designed to be used as a packing material. The sponge is composed of hydroxylated polyvinyl acetate that will increase in size within the nasal cavity and compress a bleeding vessel through rehydration with normal saline.

Several different types of absorbable nasal packing are available on the market such as Surgicel® (oxidized regenerated cellulose), Floseal® (gelatin granules and human thrombin), and Arista (plant starch). Surgicel® is notably less expensive than the other two products.^9^, ^10^

**FIGURE 1:**
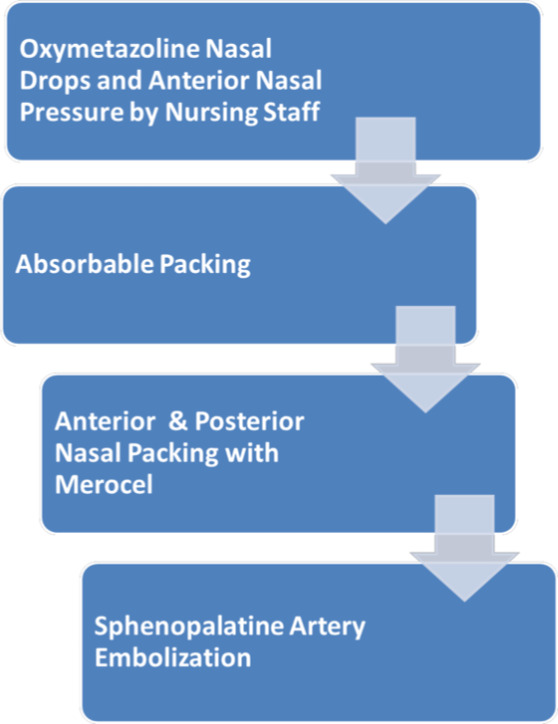
Epistaxis management for COVID‐19–positive patients

Since the COVID‐19 pandemic has commenced, our otolaryngology department has been consulted for numerous cases of epistaxis on COVID‐19–positive patients, and the four included cases serve as examples of the different ways in which the bleeding was managed in the setting of the specific disease entity. Case 4 was managed conservatively without invasive intervention as compared to cases 1, 2, and 3. This supports the algorithm in Figure 1 and the saved PPE while increasing safety of excess exposure to consulting ENT physicians. All the patients were observed for 48 hours, and no further bleeding was noted.

Based on available literature and experience before the COVID‐19 pandemic, the following guideline as shown in Figure 1 was proposed to manage epistaxis in COVID‐19–positive patients. The algorithm depicted is based around limiting viral exposure in terms of quantity of staff interaction and effective treatment of epistaxis. Subsequently, the progression of treatment is designed in a stepwise framework to first attempt conservative management via verbal instructions to nursing, with appropriate escalation thereafter should initial treatment fail.

The first step is to inform the treating nurse to apply topical oxymetazoline drops and apply local pressure application if bleeding is minimal. If not controlled after 10 minutes, the otolaryngologist should then evaluate the patient with proper personal protective equipment as described above, and pack the nose with Surgicel® Fibrillar or other absorbable packing available at their institution. As seen in cases 1, 2, and 3, absorbable packing was able to control epistaxis without the need for regular follow up contact with the patient.^11^, ^12^

Nonabsorbable nasal packing is not recommended unless other measures as shown in Figure 1 are not first successful. Merocel® packing requires removal after 48‐72 hours, and there is a higher chance of aerosolization of nasal secretions and disruption of nasal mucosa in the process of removing the nasal packs.^13^, ^14^, ^15^ The Academy of Otolaryngology‐Head and Neck Surgery has recommended several studies which show that nasal endoscopy with cautery produced a significant amount of airborne aersols.^16^ Due to these recommendations, we also would avoid nasal endoscopic control of posterior nasal bleeding with clipping or cautery of the sphenopalatine artery. Embolization may be the best option for these COVID‐19–positive patients with posterior nasal bleeding. The Detroit Medical Center Otolaryngology department is practising the above protocol, and thus far, no one from the department has had shown any COVID‐19 symptoms.

There are several limitations in this report including the use of small sample size, extrapolation of a single institutional experience, and the presence of confounding factors (aspirin intake, probable hypertension), which suggest that the origin of epistaxis is unclear.

## 5 CONCLUSION

Epistaxis management on COVID‐19–positive patients is concerning for otolaryngologists due to the highly virulent nature of COVID‐19 in respiratory droplets and nasal secretions. Authors suggest the oxymetazoline nasal drops and local pressure initially by treating nurse for about 10 minutes before considering nasal packing with preferably absorbable material to prevent COVID‐19 transmission to otolaryngology physicians.

## CONFLICT OF INTEREST

None declared.

## AUTHOR CONTRIBUTIONS

SSRB: contributed to conception and design, acquisition of data, analysis and interpretation, drafting the article, critical revision and final approval of article, and agreement to be accountable for all aspects of the work. LS: contributed to acquisition of data, analysis and interpretation, critical revision and final approval of article, and agreement to be accountable for all aspects of the work. TP: contributed to analysis and interpretation, drafting the article, critical revision and final approval of article, and agreement to be accountable for all aspects of the work.

## CONSENT STATEMENT

Published with written consent of the patient legal guardians.
